# HPLC-FLD-Based Method for the Detection of Sulfonamides in Organic Fertilizers Collected from Poland

**DOI:** 10.3390/molecules27062031

**Published:** 2022-03-21

**Authors:** Zbigniew Osiński, Ewelina Patyra, Krzysztof Kwiatek

**Affiliations:** Department of Hygiene of Animal Feedingstuffs, National Veterinary Research Institute, Partyzantów 57 Avenue, 24-100 Pulawy, Poland; zbigniew.osinski@piwet.pulawy.pl (Z.O.); kwiatekk@piwet.pulawy.pl (K.K.)

**Keywords:** sulfonamides, HPLC-FLD, liquid manure, solid manure, method validation

## Abstract

Antibacterial substances such as sulfonamides are widely used in veterinary medicine to treat many bacterial diseases. After their administration to animals, up to 90% of the initial dose of the antibiotic is excreted in the feces and/or urine, which can be applied to farmland as natural or organic fertilizers. In this work, an analytical method was developed with the use of HPLC-FLD for the detection and quantification of five sulfonamides (sulfaguanidine, sulfadiazine, sulfamerazine, sulamethazine and sulfamethoxazol) in poultry and pig feces, slurry and digestates. The method was validated according to EU requirements (Commission Decision 2002/657/EC and VICH GL49). Linearity, decision limit, detection capability, detection and quantification limits, recovery, precision, and selectivity were determined, and adequate results were obtained. Using the HPLC-FLD method for all analyzed matrices, recoveries were satisfactory (77.00–121.16%), with repeatability and reproducibility in the range of 4.36–17.34% to 7.94–18.55%, respectively. Decision limit (CCα) and detection capability (CCβ) were 33.87–67.63 and 53.36–92.00 µg/kg, respectively, and limit of detection (LOD) and limit of quantification (LOQ) were 13.53–23.30 and 26.02–40.38 µg/kg, respectively, depending on the analyte. The forty-four samples of natural and organic fertilizers were analyzed, and four samples showed sulfamethoxazole in the amount from range 158 to 11,070 µg/kg. The application of antibiotics including sulfonamides for farming animals is widespread and may lead to the development of antibiotic resistance and other environmental effects.

## 1. Introduction

Antibacterial substances are used all around the world for treatment in human therapy and veterinary medicine. For animal treatment, antibiotics are mainly administrated by three different routes: through injection, via feed with medicated feed, or via drinking water. Most pharmaceuticals are designed to be quickly excreted from the treated organism in unchanged form, or as metabolites that may still exhibit pharmacological activity [1]. After the administration of antibiotics, between 30% and 90% of the initial dose given is excreted [2]. Thus, poultry droppings, pig feces and liquid manure, used as fertilizer for agricultural land, are often contaminated with antibiotics [3].

The contribution of natural and organic fertilizers as carriers of antimicrobial environmental contaminants has gradually become more recognized globally. Studies have shown that antibiotics cannot be degraded effectively during the composting process; therefore, the practice of placing animal manure on fields used to produce food poses a risk of contaminating the soil [4].

A commonly used class of antibacterial substances are sulfonamides. Sulfonamides are a class of derivatives with p-aminobenzene sulfonamide as the basic structure. They have wide antibacterial action against gram-negative and gram-positive bacteria, and they can also act on some rickettsia and protozoa (e.g., *Toxoplasma*, *Coccidia*). Due to their low price and broad-spectrum activity to various bacteria, they are widely used to treat bacterial diseases in animal husbandry.

According to the list of veterinary medicinal products authorized on the market in the Republic of Poland, sulfadiazine and sulfaguanidine can be used in slaughter animals in the form of medicated feed, sulfamethoxazole and sulfamerazine can be used in animal drinking water, and sulfadiazine can be given by injection and in the form of tablets and granules [5]. For sulfonamides, maximum residue limits have been established in the tissues and organs of slaughter animals and in milk, the total residue content of which of all substances from the sulfonamides group should not exceed 100 μg/kg [6]. In the case of natural and organic fertilizers, there is no laboratory control of the content of antibacterial substances belonging to this group and no residue limits have been established. Therefore, after the introduction of natural or organic fertilizers on agricultural land and grasslands, the sulfonamides present in them may migrate to the soil, river water, and groundwater [7]. Antibiotics could cause hazardous ecological and health impacts to the environment and pose a potential risk to the food chain, even at low concentrations [8]. Exposure to antibiotics may cause adverse effects on the reproductive system in the early life stages of different organisms [9].

In the last dozen years, researchers have developed some methods for the detection and quantification of antibacterial substances in natural and organic fertilizers with the use of liquid chromatography techniques with different kinds of detectors: mass spectrometry, fluorescence, and UV detection [2,7,10,11,12,13,14].

Several studies analyzed specific groups of antibiotics or a wide variety of compounds from different antibiotic groups in manure or feces [10,11,12]. Xiang-Gang et al. [14] detected sulfadimidine in swine manure and sulfamethoxazole in chicken dung at concentrations ranging from 3.3 to 24.8 mg/kg and from 4.5 to 18.7 mg/kg, respectively. Zhao et al. [11] analyzed three groups of antibiotics—tetracyclines, fluoroquinolones, and sulfonamides—in cattle, pigs, and poultry feces. The authors analyzed eight sulfonamides in the feces. The determined concentrations ranged from 0.01 to 6.04 mg/kg depending on the sulfonamide and the species of the animal. The lowest concentrations were found in cattle feces samples. Martinez-Carballo et al. [10] found sulfadimidine in pig slurry at concentrations levels up to 20 mg/kg. They found also sulfadiazine in feces samples from poultry farms at the maximum concentration of 51 mg/kg from chickens and 91 mg/kg from turkeys. Karci and Balcıoğlu [7] also reported the presence of sulfachloropyridazine and sulfamethoxazole in fresh poultry manure samples at maximum concentrations of 35.53 and 3.76 mg/kg, respectively.

Other researchers also found sulfonamides in animal manure. The most often detected were sulfadimidine, sulfadiazine, and sulfamethazine at the concentration range from 1 µg/kg to about 25 mg/kg [7,13,15].

When feces, manure, liquid and solid manure, litter, and digestate from biogas plants are used as fertilizer, antibiotics can be distributed onto fields and pastures on a large scale. Little is known about concentrations and the fate of antibiotics in manure and soil. These parameters are of great importance when evaluating the role of contaminated manure in the spread of antibiotic agents into the environment, and to assess the risk of water and food contamination through this pathway. Nevertheless, manure is the source of a significant part of veterinary drug pollution in the environment and is currently not active monitored. The lack of data on the amount of antibiotic residues in manure applied to agricultural land, with or without processing, is concerning and requires an adequate risk assess-ment of environmental pollution by veterinary drugs [16].

Therefore, the objective of this work was to develop a method for the detection and quantification of sulfonamides (sulfaguanidine, sulfadiazine, sulfamerazine, sulfamethazine, and sulfamethoxazole) in natural and organic fertilizers and evaluate their occurrence in samples from pig and poultry farms and biogas plants from Poland by high-performance liquid chromatography with fluorescence detection. The aim of conducting this research was to assess the amount of sulfonamides contained in natural and organic fertilizers introduced into farmland and agricultural lands.

## 2. Results and Discussion

### 2.1. Extraction Procedure

The extraction of sulfonamides from pig and poultry manure, as well as slurry and digestates, is not a simple process; the complexity of the analyzed matrices, various states of aggregation, and a large amount of natural organic matter make extraction difficult. The extraction of antibiotics from manure solids presents an additional challenge, as more matrix interferents are associated with manure solids than manure liquids. The higher natural organic matter content in the solids provide more site sorption, resulting in the need to use harsher conditions for extraction, for example, ultrasonic extraction [17].

Sulfonamides from food, feed or feces samples are most often extracted with organic solvents such as methanol, acetonitrile, ethyl acetate, or mixtures of organic solutions with water or buffer.

Several methods of extracting sulfonamides from animal feces have been described in research publications. Zhao et al. [11] used a sequential methanol extraction to extract sulfamethoxazole, sulfadiazine, sulfanilamide, sulfamerazine, sulfadimidine, sulfamonomethoxine, and sulfachloropyridazine. Next, the extract was passed through a column filled with a sodium sulfate to remove water and eluent was concentrated under nitrogen stream. A similar procedure with methanol and sodium sulfate was used by Qiu et al. [12]; on the other hand, Haller et al. [18] used a different procedure for the extraction of sulfonamides from the slurry. The authors first adjusted the pH of the slurry to 9 with KOH, then 1 g of NaCl was added to the samples and the sulfonamides were extracted three times with ethyl acetate. Next, the extract then dried under a nitrogen stream.

In this work, the methods described above were used in the first stage of extraction optimization. The extraction methods described by Haller et al. [18] and Zhao et al. [11] did not give good results because a large amount of interfering substances were observed, which did not allow for qualitative and quantitative analysis of the analyzed sulfonamides. Moreover, the described extraction methods were based solely on drying the obtained extract without the purification step using the SPE technique.

Therefore, it was decided to use the method described by Patyra et al. [19]. The described extraction method was dedicated to the extraction of sulfonamides from feed samples. Our experiment used the extraction method described by Patyra et al. with the use of a mixture of ethyl acetate, acetonitrile, methanol (50/25/25, *v*/*v*/*v*), and solid phase extraction techniques on Strata-SCX cartridges. After SPE procedure, the supernatant was evaporated and residues were resuspended in 0.2% fluorescamine in acetone and 0.1 M sodium acetate pH = 3.5. Sulfonamides (SAs) were derivatized for 15 min in the dark and at room temperature.

This clean-up protocol gave very good results for pig and chicken feces samples. The application of the described extraction and purification method for liquid samples of slurry and digestates turned out to be useless. This was due to the presence of water in the obtained extract. Therefore, the extract obtained was diluted with a mixture of ethyl acetate/methanol/acetonitrile (50:25:25, *v*/*v*/*v*). For this purpose, 6 mL of extract were taken and diluted with 6 mL of extraction mixture; then, purification by the developed SPE technique was applied. This approach allowed for the partial elimination of water present in the sample and satisfactory results after sample purification on a strongly cation exchange with Strata-SCX cartridges.

The described procedure for the extraction and purification of sulfonamides turned out to be suitable not only for the analysis of sulfonamides in feed, but also in even more complex analytical matrices such as animal feces, liquid manure, and digestates.

### 2.2. HPLC-FLD Analysis

**Figure 1:**
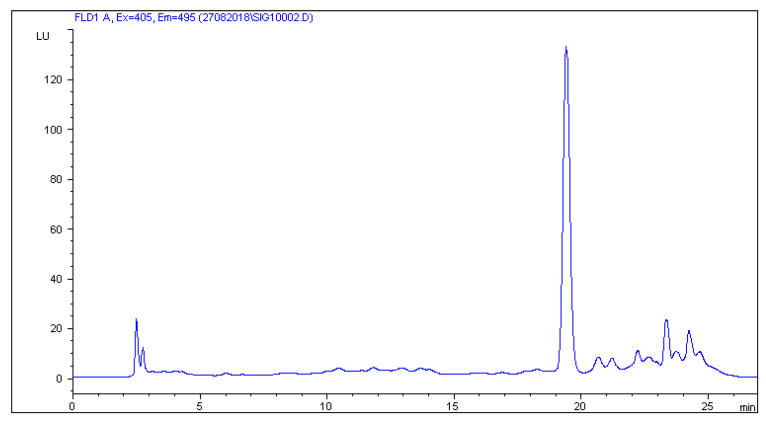
Blank sample of solid manure (pig feces).

**Figure 2:**
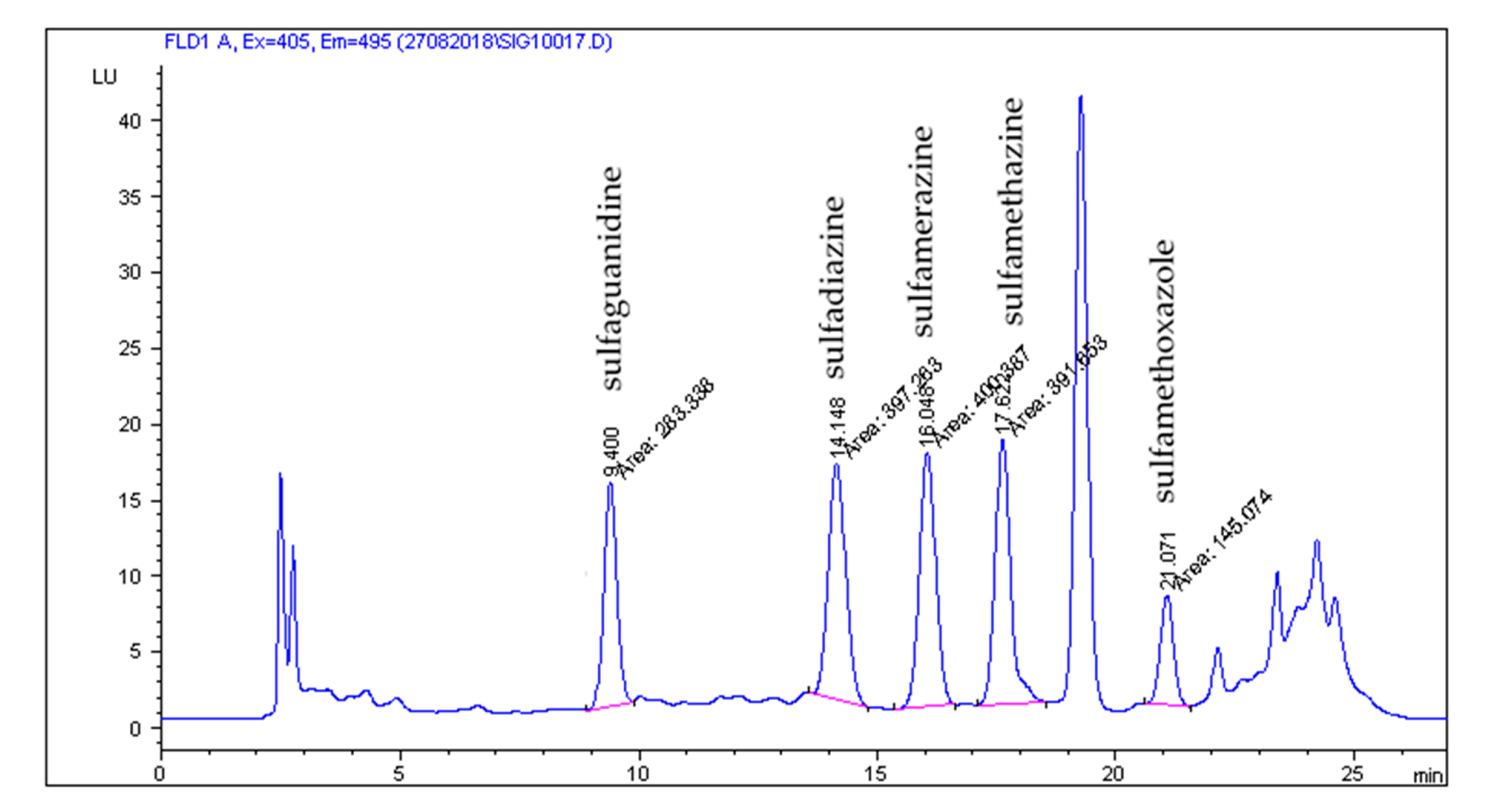
Solid manure (pig feces) sample spiked five sulfonamides at the concentration level 50 µg/kg.

**Figure 3:**
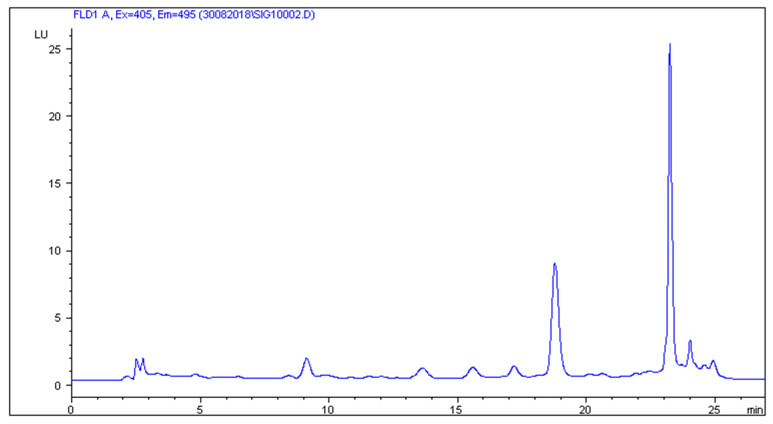
Blank sample of liquid manure.

**Figure 4:**
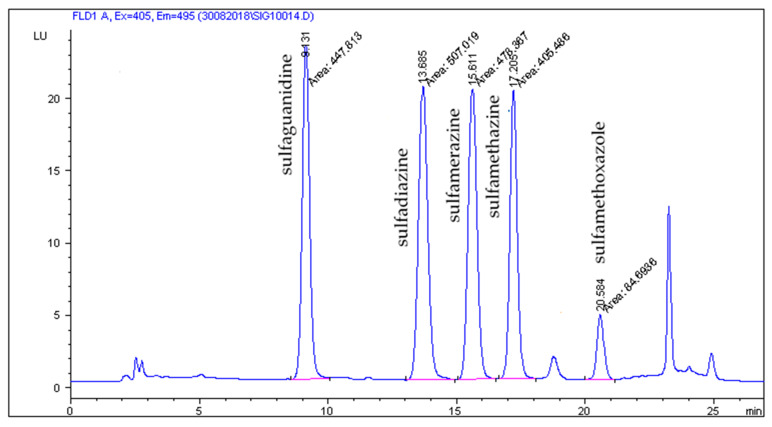
Liquid manure sample spiked five sulfonamides at the concentration level 50 µg/kg.

The HPLC-FLD method developed by Patyra et al. [19] proved useful for the separation and detection of five sulfonamides from pig and poultry feces, liquid manure, and digestates. The use of the Zorbax Eclipse XDB C18 (150 × 4.6 mm, 5 µm) column and the mobile phase consisting of 0.08% acetic acid in combination with methanol and acetonitrile and gradient elution turned out to be useful not only for the analysis of sulfonamides in feed but also in natural and organic fertilizers. The use of the chromatographic separation parameters described in Section 3.6 gave satisfactory separation results. Chromatograms of blank liquid manure, pig feces samples, and samples fortified with sulfonamides at a concentration level of 50 µg/kg are shown in Figure 1, Figure 2, Figure 3 and Figure 4.

### 2.3. Method Validation Results

The whole procedure was validated according to the requirements of the Commission Implementing Regulation (EU) 2021/808 and VICH GL49 [20,21].

Results for the method validation using the sample preparation described in Section 3.7 are summarized in Table 1 and Table 2. Matrix calibration curves were constructed using the ratio of analyte peak area versus analyte concentration for all analyzing sulfonamides. For all analytes, the linear concentration range was from 50 µg/kg to 1000 µg/kg with coefficients of determination higher than 0.98. The specificity of the method was tested by processing and analyzing 20 control pig and chicken feces and 20 liquid manure and digestate samples.

Values for the recovery of the spiked samples were in the range of 77.00–107.85% for pig and poultry feces and 81.20–121.56% for liquid manure and digestate for all analyzed sulfonamides. The intra-day and inter-day precision of the methods were evaluated at three concentration levels (50, 200, and 1000 µg/kg). For this purpose, six spiked samples at each level were prepared and analyzed. This procedure was repeated for three days in order to determine the inter-day precision. The repeatability for the target analytes was lower than 15% and 17% for pig and poultry manure and liquid manure and digestate, respectively. The within-laboratory reproducibility was lower than 19% for all analyzing matrices at all spiking levels. In the described method, both LOD and LOQ values were determined. The LOD for the sulfonamides in pig and poultry feces was 15.59–23.30 µg/kg, and for liquid manure and digestate this value was 13.53–17.93 µg/kg. The LOQ was 26.02–35.88 µg/kg and 30.21–41.97 µg/kg for all analyzing matrices. All validation parameters are shown in Table 1 and Table 2.

### 2.4. Presence of SAs in Animal Feces, Liquid Manure, and Digestate

Poultry droppings, pig feces, liquid manure, and digestate are commonly used around the world to improve soil fertility. Using these fertilizers is an economical way to dispose of the waste products generated in substantial quantities by the production of animals [22]. There is evidence indicating that natural fertilizers do not meet the minimum standards for application as an organic fertilizer; mainly due to the presence of contaminants such as antimicrobial residues, pathogens, antimicrobial resistance genes, and heavy metals, among others. Despite this, natural fertilizers continue to be used, and there are no regulations regarding its use as an agricultural fertilizer [23].

Antibiotics are frequently detected in an unchanged form in manure and slurry from animal farms. The concentrations detected vary depending on the animal species, the nature of the antibiotic, the geographical sampling area, and the type of livestock farm, ranging from ug/kg to hundreds of mg/kg. SAs are routinely used in veterinary medicine to treat a variety of bacterial and protozoa infections in cattle, swine, and poultry. Zhao et al. [11] detected sulfonamides in cattle, swine, and poultry feces samples. The concentration of sulfonamides was between 30 µg/kg for sulfaguanidine to 6.04 mg/kg for sulfadimidine. Martinez-Carballo et al. [10] reported that sulfadimidine was present in 18 of 30 liquid manure samples from pigs, at concentration levels up to 20 mg/kg. Sulfadiazine was detected in dung samples from poultry farms with maximum concentrations of 51 mg/kg in poultry feces and 91 mg/kg for turkeys. Jensen et al. [15] analyzed 34 samples of veal calve manure from the Netherlands. Out of the total of 34 samples, 33 tested positive (97%). The antibiotics most frequently found with the highest concentration were oxytetarcycline, doxycycline, flumequine, tilmicosin, and sulfadimidine. They found three sulfonamides in manure samples—sulfadizine at concentrations ranging from 1 to 65 µg/kg, sulfadimidine from 2 to 5100 µg/kg, and sulfamethazine from 1 to 7 µg/kg. Hou et al. [13] found a high concentration of SAs in animal manure samples collected in Tianjing, China. Sulfadimidine was detected in six swine manure samples and eight chicken dung samples at a concentration range of 3.3–24.8 mg/kg. Sulfamethazine was detected in two chicken dung samples at 2.3 and 5.2 mg/kg. Sulfadiazine also was detected in ranging from 4.5 to 18.7 mg/kg. Karci and Balcioglu [7] also reported the presence of sulachloropyridazine and sulfamethazine in fresh poultry manure samples at the concentrations of 35.53 and 3.76 mg/kg, respectively. Zheng et al. [24] analyzed 66 manure samples and they found SAs residues in more than 83% of the manure samples. Seven of the manure samples contained more than three SAs. Sulfamonomethoxine and sulfabenzamide were the main SAs detected in cow manure, where concentrations ranged from 2.4 to 24.4 μg/kg. In chicken manure, they detected SAs in amounts ranging from 1 to 1908 µg/kg, while in pig manure from 2 to 6099.6 µg/kg. Zheng et al. [24] found the presence of sulfadiazine, sulfamonomethoxine, sulfaquinoxaline, sulfathiazole, and sulfabenzamide in the analyzed samples. In this study, we analyzed forty-four samples from different regions of Poland. Twenty-five samples of pig feces, fifteen samples of dygestate, two samples of poultry feces, and two samples liquid manure were analyzed. In four samples (9%) sulfonamides were found, but only sulfamethoxazole. Sulfamethoxazol was found in one instance of poultry feces and in three instances of pig feces, and concentrations were between 158 and 11,070 µg/kg. Other scientists did not confirm the presence of sulfamethoxazole in natural fertilizer samples, which is different from the results obtained by us. Only Hu et al. [25] detected the presence of sulfamethoxazole in liquid manure. They analyzed liquid manure and soil samples from Tianjin, China. Moreover, they analyzed the liquid manure obtained from the animals during the summer and winter periods. The analyzed samples of liquid manure confirmed the presence of oxytetarcycline, tetracycline, chlortetracycline, chloramphenicol, lincomycin, ofloxacin, ciprofloxacin, pefloxacin, sulfamethoxazole, sulfadoxine, and sulfachloropyridazine. On the basis of the obtained results, the authors concluded that higher concentrations of antibiotics were found in the liquid manure collected from animals in winter. This was due to the more frequent need to treat animals during the winter season. Hu et al. [25] confirmed the presence of sulfonamides in liquid manure at concentrations from 0.03 mg/kg to 32.7 mg/kg, with the highest concentrations obtained for sulfadoxine. The determined concentrations of sulfomethoxazole in liquid manure ranged from 2 to 5.7 mg/kg in winter and from 0.23 to 2.0 mg/kg in the liquid manure collected in summer.

The results we obtained differed from those presented by other authors. Perhaps this is because other countries prefer to use other sulfonamides than sulfamethoxazole for therapeutic purposes.

**Figure 5:**
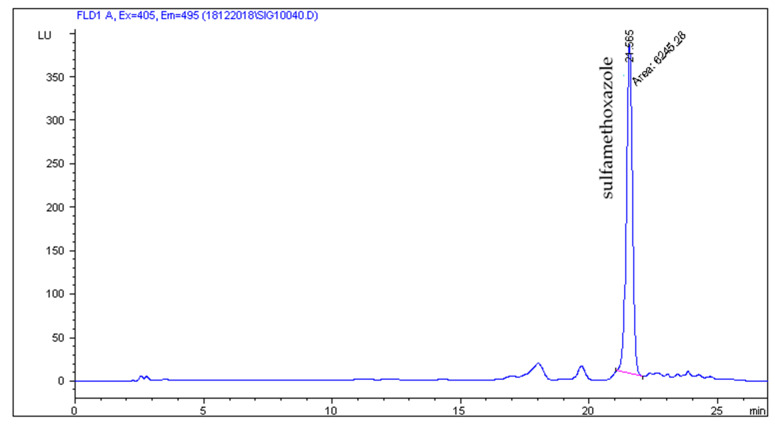
Real sample of solid manure (poultry droppings) with sulfamethoxazole—2000 µg/kg.

Our results showed that sulfonamides in animal feces samples were not detected frequently, but the concentrations were comparable to published research results by other scientists around the world. The results may also indicate that other antibiotics are used more often than sulfonamides in Poland, e.g., tetracycline antibiotics. The results of the research indicate that the presence of antimicrobial substances in natural and organic fertilizers should be monitored before introducing them to farmland. The amounts of sulfamethoxazole found in the present study could have an ecotoxic effect on the microbiota inhabiting land and surface waters, and could be absorbed by crops. Results for positive samples are shown in Table 3. Figure 5 presents an example chromatogram of a real sample of poultry feces with sulfamethoxazole.

## 3. Material and Methods

### 3.1. Chemicals and Reagents

Certified standards of sulfaguanidine, sulfadiazine, sulfamerazine, sulfamethazine, sulfamethoxazole, and fluorescamine were obtained from Sigma Aldrich (St. Louis, MO, USA). All reagents used were of analytical grade, >95% purity. Acetonitrile and methanol for HPLC were from J.T. Baker (St. Louis, MO, USA). Ethyl acetate, acetic acid and ammonia solution (25%) were purchased from POCH (Gliwice, Poland). High purity water with a resistivity of 18.2 MΩ cm^−1^ was obtained from a Milli-Q water system (Millipore, Bedford, MA, USA).

### 3.2. Reagents

Standard stock solutions of individual sulfonamides (1 mg/mL) were prepared in methanol for SGD, SDZ, SMZ and SMO. An SDZ standard was prepared by dissolving in acetonitrile. A mix solution containing all reference standards was prepared for the in-house validation in methanol at a level of 100 µg/mL and kept in the freezer no longer than 6 months.

A fluorescamine solution was prepared by weighting 20 mg of standard and dissolving it in 5 mL of acetone. The fluorescamine solution was stored in a dark glass bottle at −18 °C for less than 3 months.

Extraction solution was prepared by mixing ethyl acetate/methanol/acetonitrile in the ratio of 100:50:50 (*v*/*v*/*v*). Extraction solution was prepared fresh on the day of sample preparation.

### 3.3. Extraction

Five grams of solid or liquid manure were added to 50 mL polypropylene centrifuge tubes. Exploratory procedures were adopted from a previously reported method by Patyra et al. [19] with minor modifications. Sulfonamides were added to control samples and shaken for 15 s on a vortex mixer and then kept in the dark for 3 h for equilibration. Then, 20 mL of extraction solution was used for liquid manure/dygestate, or 25 mL of extraction solution for solid manure. Samples were shaken for 45 min on a horizontal shaker, and then centrifuged at 4000 rpm for 20 minutes at 20 °C.

After centrifugation, 6 mL of the supernatant obtained from the liquid manure or digestate was transferred to a new centrifuge tube, and 6 mL of extraction solution was added.

### 3.4. Clean-Up

For the clean-up step, the SPE apparatus and Strata-SCX cartridges (500 mg, 3 mL) were used. Solid phase extraction columns were conditioned with 5 mL of 40% acetic acid in acetonitrile; next, 12 mL (liquid manure or digestate) or 6 mL (solid manure) of samples were loaded. After percolation, the cartridges were washed with 2.5 mL of aceton, 2.5 mL of methanol, and 2.5 mL of acetonitrile. The analytes were eluted twice with 2.5 mL of a mixture of 1.25% ammonium in acetonitrile and evaporated under nitrogen flow in a water bath at 45 ± 5 °C.

Next, dry residue was resuspended in 200 µL of the fluorescamine reagent. Then, 800 µL of acetate buffer (pH = 3) was added and the solution was mixed with a vortex mixer. The sample was ready to analyze after standing for 15 min at ambient temperature in a dark place.

### 3.5. HPLC-FLD Analysis

Samples were analyzed using an Agilent Technologies 1100 liquid chromatograph (Santa Clara, CA, USA) equipped with an automatic injector, degasser system, quaternary pump with four solvent channels, a column thermostat, and fluorescence. The chromatographic separation of sulfonamides was performed using a method previously described by Patyra et al. [19]. The Zorbax Eclipse XDB (150 × 4.6 mm, 5 µm) column from Agilent Technologies (Santa Clara, CA, USA) protected by a RP18 guard column (4.0 × 3.0 mm, 5 µm) from Phenomenex (Torrance, CA, USA) was used for sulfonamides separation. The mobile phase consisted of 0.08% acetic acid in water (*v*/*v*; phase A), acetonitrile (phase B), and methanol (phase C). The gradient profile is shown in Table 4. The flow rate was 0.6 mL/min and the column thermostat was set at 25 °C. The injection volume was 40 μL. The excitation and emission wavelengths for all analyzed sulfonamides were 405 and 495 nm, respectively, and total run time was 27 minutes for each injection.

### 3.6. Validation Studies

The procedure was validated according to the Commission Implementing Regulation (EU) 2021/808 and the VICH GL49 “Guidance for Industry document regarding: Validation of analytical methods used in residue depletion studies” [20,21]. The method characteristics such as: linearity, recovery, repeatability, reproducibility, decision limit, and detection capability, in addition to limit of detection and limit of quantification, were specified.

Six points of matrix-matched calibration curves that spiked at the levels 0, 50, 100, 200, 500 and 1000 µg/kg were obtained by plotting the response of respective analyte peak area ratio versus the analyte concentration.

The limit of detection and limit of quantification were evaluated statistically as 3 and 10 times of the standard deviation of the signal of blank solution for each sulfonamide, respectively. Decision limit and detection capability were determined according to the Commission Implementing Regulation (EU) 2021/808 for substances with no permitted limit. The decision limit was calculated with a statistical certainty of 1 − α (α = 1%) whereas detection capability was calculated with a statistical certainty of 1 − β. Detection capability was calculated as decision limit plus 1.64 times the corresponding standard deviation (β = 5%). Selectivity/specificity of the method was tested by analyzing 20 blank feces samples and 20 blank liquid manure samples to verify the absence of potential interfering endogenous compounds at the target analyte retention times.

Intra-day precision was assessed by comparing the results of six replicates prepared the same day at three different concentrations (50,200 and 1000 µg/kg). The procedure was repeated to determine inter-day precision by comparing results from samples prepared and analyzed on three different days. Coefficients of variation (CV, %) and standard deviations (SD) were calculated for each level. Percentage recoveries were calculated as the 100.00 × measured content/fortification level.

### 3.7. Fertilizer Samples

A total of forty-four fertilizer samples were collected from pig and poultry farms and biogas plants in Poland. In this study, 25 samples of solid manure from pigs, 2 samples of poultry droppings, 2 samples of liquid manure from pig farms, and 15 digestate samples from biogas plant were analyzed. Before analysis, all samples were stored in plastic containers and refrigerated at −20 °C until analysis.

## 4. Conclusions

A sensitive and robust method for the determination of five sulfonamides in liquid and solid manure and digestate has been developed. Sample preparation was performed using SPE followed by analysis using HPLC-FLD. Forty-four fertilizer samples were analyzed with the developed method in four samples, and the presence of sulfamethoxazole with a maximum concentration exceeding 11 mg/kg was found. The content of sulfamethoxazole in natural fertilizer revealed that the wide application of antibiotics to animal breeding is becoming a great problem. Thus, supervision on the application of antibiotics in stockbreeding should be strengthened. In addition, due to the high concentration of antibiotics in feces, it should be properly treated before being applied the soil.

## Figures and Tables

**Table 1:** Validation results for liquid manure and digestate.

Analyte	Recovery (%)			Repeatability (%)			Within-Laboratory Reproducibility (%)			Limit of Detection (µg/kg)	Limit of Quantification (µg/kg)	Decision Limit (µg/kg)	Detection Capability (µg/kg)
	Concentration Levels (µg/kg)			Concentration Levels (µg/kg)			Concentration Levels (µg/kg)						
	50	200	1000	50	200	1000	50	200	1000				
Sulfaguanidine	103.20	119.63	81.20	13.68	11.58	9.20	16.03	11.50	8.77	13.53	26.02	33.87	53.36
Sulfadiazine	99.53	117.66	82.96	14.08	11.70	9.88	16.84	10.79	8.51	14.14	29.27	38.78	60.15
Sulfamerazine	101.07	120.94	82.53	12.57	8.28	8.12	16.48	9.83	7.94	17.93	34.88	45.58	65.69
Sulfamethazine	100.93	121.16	81.76	14.75	4.36	9.24	13.42	9.73	8.80	15.11	31.33	38.44	60.28
Sulfamethoxazole	90.53	115.14	84.93	9.78	12.30	7.64	18.55	13.76	6.83	16.71	35.88	53.80	73.12

**Table 2:** Validation results for pig and poultry feces.

Analyte	Recovery (%)			Repeatability (%)			Within-Laboratory Reproducibility (%)			Limit of Detection (µg/kg)	Limit of Quantification (µg/kg)	Decision Limit (µg/kg)	Detection Capability (µg/kg)
	Concentration Levels (µg/kg)			Concentration Levels (µg/kg)			Concentration Levels (µg/kg)						
	50	200	1000	50	200	1000	50	200	1000				
Sulfaguanidine	77.00	86.16	80.42	14.59	11.26	15.41	13.52	15.29	11.48	17.11	30.21	53.61	71.37
Sulfadiazine	96.86	108.71	80.04	11.77	10.64	14.91	11.55	13.61	14.30	15.59	33.30	33.31	56.67
Sulfamerazine	78.80	101.43	79.14	12.59	11.89	16.11	18.82	14.48	14.46	20.75	40.38	34.04	54.02
Sulfamethazine	96.46	107.85	77.34	15.27	12.41	17.34	14.54	14.78	15.57	16.62	34.00	37.19	59.56
Ssulfamethoxazole	125.63	91.89	85.77	15.45	13.35	10.39	15.94	16.54	12.37	23.30	41.97	67.63	92.00

**Table 3:** The analytes and their concentrations detected in real samples.

	Analyte (µg/kg)					
	Sulfaguanidine	Sulfadizine	Sulfamerazine	Sulfamethazine	Sulfamethoxazol	Standard Deviation
**Pig feces**						
**S1**	-	-	-	-	11,070	245
**S2**	-	-	-	-	6877	313
**S3**	-	-	-	-	158	18
**Poultry feces**						
**S4**	-	-	-	-	2000	90

**Table 4:** Gradient elution of sulfonamides with HPLC-FLD detection [19].

Time (min)	Phase A 0.08% Acetic Acid in Water (%)	Phase B Acetonitrile (%)	Phase C Methanol (%)
0	48	10	42
10	48	10	42
15	41	10	49
17	41	10	49
20	18	40	42
22	48	10	42
27	48	10	42

## Data Availability

Not applicable.

## Sample Availability

Samples of the compounds are not available from the authors.
